# Molecular epidemiology of *Giardia duodenalis* infection in humans in Southern Ethiopia: a triosephosphate isomerase gene-targeted analysis

**DOI:** 10.1186/s40249-018-0397-4

**Published:** 2018-03-05

**Authors:** Mengistu Damitie, Zeleke Mekonnen, Tadesse Getahun, Dante Santiago, Luc Leyns

**Affiliations:** 1 Department of Environmental Health Sciences and Technology, Jimma University, Jimma, Ethiopia; 2 School of Medical Laboratory Sciences, Jimma University, Jimma, Ethiopia; 3 Department of Biology, Faculty of Science and Bioengineering Sciences, Vrije Universiteit Brussel, Brussels, Belgium

**Keywords:** *Giardia duodenalis*, Assemblage, Molecular epidemiology, Risk factors, Triosephosphate isomerase gene, Ethiopia

## Abstract

**Background:**

*Giardia duodenalis* is a species complex consisting of multiple genetically distinct assemblages. The species imposes a major public health crisis on developing countries. However, the molecular diversity, transmission dynamics and risk factors of the species in these countries are indeterminate. This study was conducted to determine the molecular epidemiology of *G. duodenalis* infection in asymptomatic individuals in Southern Ethiopia.

**Methods:**

From March to June 2014, fresh stool samples were collected from 590 randomly selected individuals. Socio-demographic data were gathered using a pre-tested structured questionnaire. The genotyping was done using triosephosphate isomerase gene-based nested polymerase chain reaction and DNA sequencing. The genetic identity and relatedness of isolates were determined using the basic local alignment search tool and phylogenetic analysis. Risk factors associated with *G. duodenalis* infection were analysed using binary and multinomial logistic regression models.

**Results:**

The results showed that 18.1% (92/509) of the study subjects were infected by *G. duodenalis*. Among the isolates, 35.9% (33/92) and 21.7% (20/92) were sub-typed into assemblages A and B, respectively, whereas 42.4% (39/92) showed mixed infections of A and B. Most of the assemblage A isolates (94%,31/33) were 100% identical to sequences registered in GenBank, of which the majority belonged to sub-assemblage AII. However, the high genetic variability and frequency of double peaks made sub-genotyping of assemblage B more problematic and only 20% (4/20) of the isolates matched 100% with the sequences. The risk factors of age (*P* = 0.032) and type of drinking water source (*P* = 0.003) both showed a significant association with the occurrence *G. duodenalis* infection.

**Conclusions:**

This study established the endemicity of *G. duodenalis* in Southern Ethiopia. Infection with assemblage A was more frequent than with assemblage B, and the rate of infection was higher in children and in municipal/tap and open spring water consumers than the other groups. Sub-typing of assemblage B and determining the origin of double peaks were challenging. The present study confirms the need for further inclusive studies to be conducted focusing on sub-types of assemblage B and the origin of heterogeneity.

**Electronic supplementary material:**

The online version of this article (10.1186/s40249-018-0397-4) contains supplementary material, which is available to authorized users.

## Multilingual abstracts

Please see Additional file 1 for translations of the abstract into the six official working languages of the United Nations.

## Background

*Giardia duodenalis* is a flagellated protozoan parasite that can live in humans and animals, and affects the public health of many developing countries [1, 2], including Ethiopia [3, 4]. Eight genetically distinct genotypes (assemblage A to H) of *G. duodenalis* have been identified [5, 6]. Two of the assemblages (A and B) have broad host ranges [6, 7] and parasitise humans [8, 9], marine vertebrates [6] and certain other mammals [10, 11]. The remaining six assemblages (C to H) are host-specific and infect animals [6, 11], although assemblages C [1, 12] and E [13] have been reported from human isolates.

The parasite has a single host life cycle that alternates between two different forms (trophozoite and cyst stages), which are responsible for the occurrence and transmission of the disease, respectively. The cyst stage is stable and resists a range of environmental conditions and remains viable for months, preferably in cold and wet environments. Contamination of potable water with the stool of infected individuals or domestic animals and consumption of raw vegetables are known to ease the transmission of *G. duodenalis* [14]. Rapid filtration and water chlorination facilities are insufficient to disinfect the cysts and consequently there is a consistent presence of cysts on the surface of the water, which is used for human consumption [15].

Ingestion of cysts paves the way for trophozoites to emerge from the cysts and attach to the small intestinal mucosa of the host, resulting in asymptomatic to symptomatic infections. Symptomatic infections may vary in severity and cause diarrhoea, abdominal pain, vomiting, nutrient malabsorption, weight loss, and impaired growth and development in children [5, 16]. The evolutionary genetic recombination among isolates may impact the variability of the disease, as it reinforces the parasitic resistance to host immunity and anti-protozoan treatments [17].

Despite several studies on this subject, the epidemiology of *G. duodenalis* is still complex [10, 18, 19], as there is high polymorphism among isolates [20, 21]. In most developing countries, different permissible conditions exacerbate the transmission of *G. duodenalis* [10, 16], however, there is still a lack of understanding of its molecular epidemiology. In such areas, molecular studies are useful to better understand the significance of the parasite.

Despite the presence of morphologically indistinguishable genotypes and complexity of *G. duodenalis*, identification and reporting systems in Ethiopia mainly rely on light microscope. Therefore, the present study aimed at elucidating the epidemiology of *G. duodenalis* infection in Southern Ethiopia by using a molecular platform method.

## Methods

### Study area

This study was conducted in the Southern Nations, Nationalities and Peoples’ Region of Ethiopia, which is interchangeably known as Southern Ethiopia. According to the regional and geographical classification system of the country, the region covers about 10% of the landmass of Ethiopia, with an altitude range of 376–4207 m above sea level. It is home to 5% of the country’s population and consists of 13 administrative zones, located in 4°, 43′–8°, 58’ N latitude and 34°, 88′–39°, 14′ E longitude, with annual rainfall and temperature ranges of 500–2200 mm and 15–30 °C, respectively. The region has three types of climatic zones, which are locally known as *dega*, *woina-dega* and *kolla*. All three climatic zones were included in this study. *Dega* denotes a temperature range of 10–20 °C for 4–12 months/year. *Woina-dega* denotes temperatures above 20 °C for 4–11 months/year and 10–20 °C for the rest of the year. *Kolla* denotes annual and monthly average temperatures of above 20 °C.

People in this region get potable water from different sources, ranging from treated to untreated sources of municipal/tap, unprotected open spring, enclosed/spring box and river/stream water.

### Study design

This was a cross-sectional epidemiological study conducted from March to April 2014 among 509 individuals living in 21 districts of Southern Ethiopia.

The study subjects were asymptomatic individuals who lived in the region for at least one month prior to the study commencing and who did not experience any protozoa-related symptoms or underwent chemotherapy. The sample size was determined using the single population proportion formula, with participants who fulfilled the selection criteria being included in the study.

The study area in the region was selected using multi-stage random sampling techniques. Seven zones from the region and three districts from each zone were randomly selected. In each district/village, the households were selected using systematic sampling techniques, with an individual per household randomly selected. In brief, the households in each district or village were listed in a random order and the interval size or gap needed between sampling households was calculated by dividing the total number of households in each district or village to the sample size needed for each district or village. To pick up the household where to start sampling, an element of randomness was introduced and an integer from 1 to the calculated interval size was randomly selected using lottery method (different interval sizes were used to each district/village), then starting from the first household, every household found at each interval size was selected. The names of each household member were written on slips of paper, which were folded and mixed in a container and drawn as a lottery, with the drawer blindfolded.

The participants were then categorised into three age groups: < 5 years, 5–14 years and > 14 years. As it has good biological, epidemiological and statistical properties, and historical consistent with the classical categories of different prevalence rates of the parasite, children younger than 14 years were classified into two groups as done in a previous study in Ethiopia [4]. The participants’ socio-demographic data was gathered using a pre-tested structured questionnaire and stool samples were obtained and processed, as outlined in the next sections.

### Structured questionnaire

The questionnaire was pretested on a small number of respondents in the population being studied. Reliability and validity of the questionnaire was achieved by repeating the questions/interview with a small percentage of the respondents that were chosen randomly. To assure these, the selected respondents were interviewed and their responses were documented and the questions (same questions) were rephrased and the interviews were repeated, then each response was investigated and comparable responses were maintained. Prior to the administration of the questionnaire, the consent or assent (in the case of children) form of the study was explained and delivered to the study participants or head of the household. Once the basis and procedures of the study were understood and agreed upon, the head of the household or a designee signed the informed forms. Participants’ socio-demographic data (name, age, sex, and residence), types and climatic zones of potable water sources, and conditions of water storage were gathered using a pre-tested structured questionnaire.

### Stool sample collection

About two grams of fresh stool sample was obtained from each asymptomatic individual and preserved in 70% ethanol [22] in sterile dry stool containers labelled with codes corresponding to the individuals.

### DNA extraction and genotyping of *G. duodenalis* isolates

The stool sample (300 μl) was washed three times in sterile distilled water, resuspended and transferred into a microcentrifuge tube and centrifuged for 2 min at 13000 rpm, and then the supernatant was discarded. The sediment was resuspended in 300 μl sterile distilled water and centrifuged three more times to remove the ethanol.

The genomic DNA was extracted using the QIAamp Fast DNA Stool Mini Kit (QIAGEN, Inc., Hilden, Germany), as according to the manufacturer’s instructions. The extracted DNA was eluted in 200 μl of buffer ATE and the DNA was stored at − 20 °C for later downstream application.

Identification and genotyping of *G. duodenalis* was done using triosephosphate isomerase (TPI) gene fragment-based nested polymerase chain reaction (PCR) assay. With minor modification of a previous work [2], the external primers – TPI-FW1 (5’-CAGAAAATAAATIATGCCTGCTC-3′) and TPI-RV1 (5’-CAAACCTTITCCGCAAACC-3′) – that amplify 618 base pair (bp) fragment of the gene, and internal primers – TPI-FW2 (5’-CCCTTCATCGGIGGTAACTTCAA-3′) and TPI-RV2 (5’-ACATGGACITCCTCTGCCTGCTC-3′) –that amplify 557 bp fragment of the gene were respectively used in the primary and secondary PCR reactions. The specificity and efficiency of the primers were determined using primer design software, Gene (http://www.ncbi.nlm.nih.gov/gene/). The PCR conditions were also optimised using confirmed *G. duodenalis* positive samples.

The primary PCR amplification was done in 50 μl reaction mixture containing 5 μl DreamTaq™ Buffer (10×), 1 μl dNTPs mix (10 mmol/L each), 1 μl GI-TPI-FW1 and 1 μl GI-TPI-RV1 (10 μmol/L each), 0.25 μl (5 units) of DreamTaq™ DNA Polymerase (Fermentas, Lithuania), 0.25 μg/μl of genomic DNA and nuclease-free water. Amplification was carried out in 30 cycles, each consisting of: 95 °C for 45 s, 55 °C for 45 s and 72 °C for 1 min, with an initial hot start at 95 °C for 5 min and a final extension for 7 min. The secondary PCR amplification was carried out similarly, except that 4 μl of the primary PCR product was used and the final extension was prolonged to 10 min. The final PCR products were identified using 1.5% agarose gel electrophoresis at 110 v for 55 min. In every reaction, *G. duodenalis* DNA (positive control) and nuclease-free water (negative control) were included.

### Genotyping of *G. duodenalis* assemblages

Assemblage genotyping of the positive PCR products was determined by amplifying assemblage-specific TPI gene fragments.

Amplification for assemblage A-specific 332 bp of the gene fragment was carried out using forward primer TPI-FW (5’-CGCCGTACACCTGTCA-3′) and reverse primer TPI-RV (5’-AGCAATGACAACCTCCTTCC-3′) [23]. Amplification was done in 25 μl reaction mixture containing 2.5 μl DreamTaq™ Buffer (10×), 0.5 μl dNTPs mix (10 mmol/L each), 0.5 μl FW and 0.5 μl RV primers (10 μmol/L each), 0.25 μl of DreamTaq™ DNA Polymerase (5 units) (Fermentas, Lithuania), 2.5 μl of the primary PCR product and nuclease-free water. A total of 35 cycles was realised, each consisting of: 94 °C for 45 s, 64 °C for 45 s and 72 °C for 45 s, with an initial hot start at 94 °C for 10 min and a final extension for 5 min.

Assemblage B-specific 400 bp gene fragment was amplified using forward primer TPI-FW (5’-GTTGTTGTTGCTCCCTCCTTT-3′) and reverse primer TPI-RV (5’-CCGGCTCATAGGCAATTACA-3′) [24]. The amplification conditions were similar to assemblage A, except that the annealing temperature was lowered to 62 °C. The final PCR products were electrophoresed in 1.5% agarose gel at 110 v for 55 min.

### DNA sequencing and phylogenetic analysis

The final positive PCR products were sequenced using the corresponding primers used for PCR amplification. Sequencing was carried out using a capillary sequencer, 3730xl DNA Analyzer (Applied Biosystems, CA, USA), in combination with ABI PRISM® BigDye™ Terminator Cycle Sequencing Kits (Applied Biosystems, CA, USA). Sequencing was carried out at the VIB Genetic Service Facility, University of Antwerp, Belgium.

The pre- and post-sequencing processes were automated on a robotic platform consisting of Biomek® FX and NX instruments (Beckman Coulter, Fullerton, CA, USA). The chromatograms and sequences generated from this study were viewed and assembled using the BioEdit Sequence Alignment Editor Program version 7.2.5 (www.mbio.ncsu.edu/bioedit/bioedit.html). The consensus sequences were compared with sequences registered in GenBank, using the basic local alignment search tool (BLAST) (www.ncbi.nlm.nih.gov/blast). Genotyping of sub-assemblages was determined based on sequence homology (100% identity) of the isolates with sequences in GenBank.

The neighbour-joining statistical analysis was computed using MEGA version 7 software (http://www.megasoftware.net/), based on the Tamura-Nei model (TN93 + *G* + *I*) and represented by the bootstrap tree inferred from 1000 replicates. A discrete gamma distribution (shape parameter = 0.41) was used to model evolutionary rate differences among sites. Nucleotide substitution models for the data were determined using the Bayesian information criterion and corrected Akaike information criterion. Discrete gamma distribution (*+G*) with five-rate categories was used to evaluate goodness-of-fit of the models. The evolutionary rate variation among sites and section of evolutionary invariant sites (*+I*) were assumed and the phylogeny result was tested using the bootstrap and branch length test methods.

The representative sequence of assemblages A (KT728546.1), B (EU781015.1), C (AY228641.1), D (DQ246216.1), E (EU272157.1), F (AF069558.1) and G (AY228640.1) were included for comparison analysis. In addition, sub-assemblage A reference sequences – 07JTPI (KT728546.1), AIIGRW (KF963577.1), BSWAII (KF963567.1) and BRW AII (KF963573.1) – and sub-assemblage B reference sequences – BI (HQ397719.2), BIV (LO2116.1), BV (HQ666895.1), BVII (HQ666897.1), Hole H13 (KT948108.1), Swemon 200 (EU781015.1) and HS98 (KC632554.1) – were included. For further genetic variation analysis, *G. muris* (AF069565.1), *G. ardeae* (AF069564.1) and *G. microti* (AY228649.1) were also included. In the phylogenetic analysis, sequences of isolates that did not exhibit mixed assemblages were used.

### Data analysis

The data was statistically analysed using binary and multinomial regression models. The significant effect of the possible risk factors with respect to *G. duodenalis* test outcome was determined using binary logistic regression model and the Hosmer–Lemeshow test (*P-*value > 0.05).

The significance of each risk factor for assemblage infection was determined by means of univariate multinomial logistic regression model and likelihood ratio test (*P*-value > 0.05). All results were interpreted using odds ratios, 95% confidence intervals and significance level (*P-*values < 0.05). The statistical analysis was carried out using SPSS® Statistics program, version 20 (IBM Corp. USA).

## Results

### Molecular characterisation of *G. duodenalis* isolates

Of the 509 stool samples subjected to amplification and genotyping, 18.1% (92/509) isolates generated the expected band size of 557 bp of *G. duodenalis* TPI gene fragments. Among the isolates, assemblage-specific genotyping analysis showed that 35.9% (33/92) and 21.7% (20/92) were assemblages A and B, respectively. The remaining 42.4% (39/92) isolates showed the concurrent existence of assemblages A and B.

All but two (94%, 31/33) of assemblage A isolates showed 100% identity with sequences registered in GenBank. Among them, 19 were identical to sub-assemblage 07JTPI (KT728546.1), six were identical to BRW_aug’11 AII (KF963573.1), five were simultaneously identical to BSW_mar’11 AII (KF963567.1) and GRW_jul’11 AII GRW (KF963577.1), and one was identical to SW16.3 t (KT124816.1).

Among assemblage B isolates, only 20% (4/20) of the isolates showed 100% identity with sequences registered in GenBank. Two isolates were identical to isolate Swemon 200 (EU781015.1) obtained from a vervet monkey in Sweden, one was identical to sub-assemblage HS98 (KC632554.1) and one was identical to sequences (KT948108.1) and some other sequences. The remaining 16 assemblage B isolates and all mixed isolates showed 96–99% identity with sequences registered in GenBank.

**Fig. 1:**
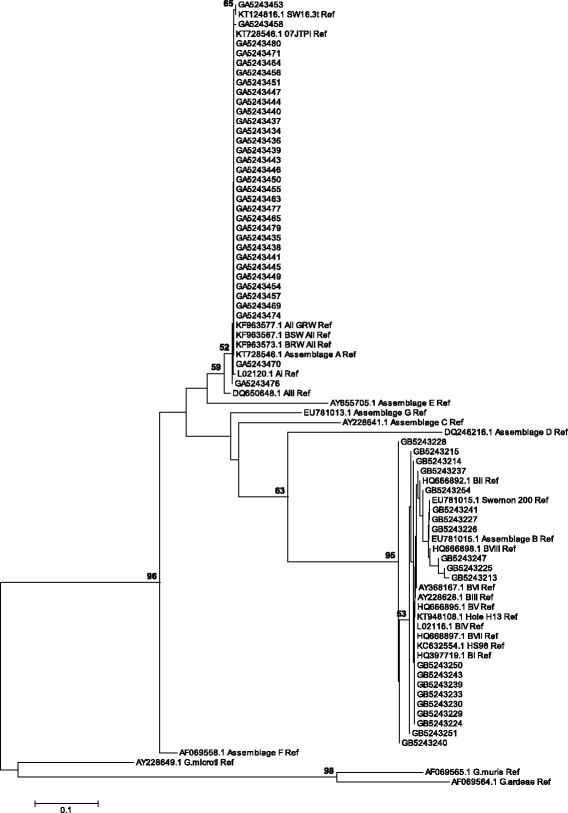
Phylogenetic tree of *G. duodenalis* assemblages based on the nucleotide sequences of TPI gene retrieved from this study compared with reference sequences from GenBank. The tree was constructed using the neighbour joining method. The evolutionary distances were computed using the Tamura-Nei method, implemented in MEGA version 7. Bootstrap values obtained from 1000 replicates are indicated next to the branches. Only bootstrap support values > 50% are shown. The scale bar indicates nucleotide substitutions per site

The neighbour-joining tree also clustered most assemblage A sequences with the reference sequences of sub-assemblage AII, however, assemblage B isolates were widely distributed within a cluster and formed tree sub-clusters. The reference sequence of assemblages C to G were separately placed, *G. muris* and *G. ardeae* formed a sub-cluster, and *G. microti* was an out-group (Fig. 1).

### Association of *G. duodenalis* infection with age and sex

Out of 509 individuals (247 males and 262 females) participated in this study, 98 were in the < 5 age group, 150 in the 5–14 age group and 261 in the > 14 age group old. The occurrence of *G. duodenalis* assemblages among different age groups and sex is indicated in Table 1.

**Table 1:** Occurrence of *G. duodenalis* infection according to individuals’ age and sex

Variables	Stool samples		Assemblage (% from positive individuals)		
	No. examined	No. positive (%)	A (%)	B (%)	A and B (%)
*Sex*					
Male	247	43 (17.4)	15 (34.9)	7 (16.3)	21 (48.8)
Female	262	49 (18.7)	18 (36.7)	13 (26.5)	18 (36.7)
*Age group (years)*					
< 5	98	24 (24.5)	9 (37.5)	3 (12.5)	12 (50.0)
5–14	150	33 (22.0)	8 (24.2)	9 (27.3)	16 48.5)
>14	261	35 (13.4)	16 (45.7)	8 (22.9)	11 (31.4)
Total	509	92 (18.1)	33 (35.9)	20 (21.7)	39 (42.4)

Age of individuals had a significant effect on the occurrence of *G. duodenalis* (*P* = 0.032). Among them, 24.5% (24/98) and 22.0% (33/150) of children in the age group of < 5 (*P* = 0.049) and 5–14 years (*P* = 0.017) were significantly infected than the age group of > 14 years (13.4%, 35/261), respectively. The estimated odds for individuals in the age groups of < 5 and 5–14 years to be infected by *G. duodenalis* were 1.824 and 1.937 times higher, respectively, than the estimated odds for individuals in the age group of > 14 years to be infected. However, the effect of age on the occurrence of assemblages was insignificant due to scattered data.

Sex showed no significant association with the occurrence of *G. duodenalis* (*P* = 0.542). Of them, males (17.4%, 43/247) and females (18.7%, 49/262) were infected. Infections with assemblages A, B and both assemblages were found in 34.9% (15/247), 16.3% (7/247) and 48.8% (21/247) of males and 36.7% (18/262), 26.5% (13/262) and 36.7% (18/262) of females, showing no association with assemblages A (*P* = 0.702), B (*P* = 0.229) or mixed infections (*P* = 0.557), respectively.

### Association of *G. duodenalis* infection with potable water sources and environments

The occurrence of *G. duodenalis* assemblages with respect to drinking water sources, climatic zones of water sources and individuals’ place of residence is summarised in Table 2.

**Table 2:** Occurrence of assemblages associated with *G. duodenalis* infection with respect to water source type, climatic zone of water source and individuals’ place of residence

Variables	Stool samples		Assemblage (% from positive individuals)		
	No. examined	No. positive (%)	A (%)	B (%)	A and B (%)
*Water source types*					
Open spring	127	33 (26.0)	12 (36.4)	6 (18.2)	15 (45.5)
Spring box	118	17 (14.4)	7 (41.2)	2 (11.8)	8 (47.1)
Municipal/tap	151	34 (22.5)	12 (35.3)	9 (26.5)	13 (38.2)
River	113	8 (7.1)	2 (25.0)	3 (37.5)	3 (37.5)
*Climatic zone of water sources*					
*Dega*	114	30 (26.3)	13 (43.3)	4 (13.3)	13 (43.3)
*Woina dega*	203	30 (14.8)	7 (23.3)	9 (30.0)	14 (46.7)
*Kolla*	192	32 (16.7)	13 (40.6)	7 (21.9)	12 (37.5)
*Climatic zone of individuals residence*					
*Dega*	113	29 (25.7)	12 (41.4)	4 (13.8)	13 (44.8)
*Woina dega*	196	30 (15.3)	7 (23.3)	9 (30.0)	14 (46.7)
*Kolla*	200	33 (16.5)	14 (42.4)	7 (21.2)	12 (36.4)
Total	509	92 (18.1)	33 (35.9)	20 (21.7)	39 (42.4)

Drinking water source had a significant effect on the occurrence of *G. duodenalis* (*P* = 0.003). Among different water source users, 26.0% (33/127) and 22.5% (34/151) of individuals who were consuming open spring water (*P* = 0.001) and municipal/tap water (*P* = 0.015), respectively showed higher infection rates than individuals (7.1%, 8/113) who were consuming river water. The estimated odds of open spring and municipal/tap water consumers being infected by *G. duodenalis* were 4.612 and 3.025 times higher, respectively, than the estimated odds of river water consumers being infected. The effect of drinking water sources on the occurrence of assemblages in individuals was insignificant due to sparseness of the data.

Individuals who were consuming water sources located in *dega* (26.3%, 30/114), *woina-dega* (14.8%, 30/203) and *kolla* climatic zones (16.7%, 32/192) were infected by *G. duodenalis*, showing no significant differences among sources (*P* = 0.287). Infections were also found in 25.7% (29/113) of *dega,* 15.3% (30/196) of *woina-dega* and 16.5% (33/200) of *kolla* inhabitants, showing no significant differences in climatic zones of individuals residence (*P* = 0.411).

## Discussion

### Molecular epidemiology of *G. duodenalis* infection

This study is one of the few molecular studies conducted in Southern Ethiopia, which generated data on the molecular diversity of *G. duodenalis* infection.

From the different genetic markers used to analyse the genetic diversity of *G. duodenalis*, this study used the TPI gene, as high genetic heterogeneity is displayed at its locus [25], and found 18.1% overall prevalence of *G. duodenalis* in asymptomatic individuals. This finding is compatible with a study done in asymptomatic individuals in Oromia Special Zone, Central Ethiopia, in which the prevalence was 16.8% [4]. Previous studies conducted in Malaysia [14, 26] and Brazil [27] likewise reported 16–18% prevalence rates of *G. duodenalis*. A study conducted in rural Malaysia also reported a 22.2% *G*. *duodenalis* prevalence rate [28], while a study in Uganda curiously reported a much high prevalence rate (40.7%) [29]. The study in Uganda suggested that the high prevalence of *G. duodenalis* was due to the occurrence of cross-species transmission of multiple *G. duodenalis* assemblages (reverse zoonotic transmission) in the area, where people, livestock and primates overlap in their use of habitat.

This study showed that all *G. duodenalis* infections were caused by assemblages A and B or combinations of these assemblages only, hence the study found out that assemblages A and B are the main causes of *G. duodenalis* infection in the region. Although previous studies have reported assemblages C and E from human isolates, the present findings do not show the presence of these assemblages and are consistent with other studies done in Brazil [1], Ethiopia [4] Malaysia [14, 26] and France [30]. Consistent with previous studies [13, 18, 31], infection with assemblage A was more prevalent than infection with assemblage B. However, the present study contradicts findings of other studies in Egypt [12] and Belgium [24] that showed a higher prevalence of assemblage B than assemblage A. Moreover, the study done in Oromia Special Zone, Ethiopia [4] (which is adjacent to the present study area) showed the predominance of assemblage B. The two studies mainly differ in the ages of their study subjects, genotyping markers they used and time/period of data collection; hence the variation of findings between the studies could lie in these factors.

The large percentage of mixed infections also reflects the complex circulation of the parasite in this region and exposure of humans to multiple sources. This finding is supported by a study [32], which reported a higher prevalence of mixed infections in developing countries than in developed ones. According to a study done in England [9], the degree of variability and number of different sub-types differ among loci, where mixed assemblage-infected isolates genotyped at TPI, βg and GDH loci resulted in inconsistent genotypic patterns. Therefore, the genotyping marker differences between the studies might also contribute to the variations in their findings. It indicates that genotyping of *G. duodenalis* at a single locus is insufficient to make a rational inference, thus a more thorough multilocus-based molecular characterisation is necessary.

### Sub-genotypes of *G. duodenalis* infection

In this study, both BLAST and phylogenetic analyses showed the genetic homogeneity of assemblage A isolates and genetic heterogeneity of assemblage B isolates. Except for a discrepancy of one isolate (GA5243476), both analyses showed similar results and grouped most isolates of assemblage A into sub-assemblage AII and one isolate (GA5243453) into SW16.3t (KT124816.1). The tree topology also provided evidence on the low sequence divergence of assemblage A isolates. However, one isolate that had 100% identity with the sequence 07JTPI (KT728546.1) in the BLAST analysis showed variation from all isolates in the phylogeny. Even though sub-assemblage AII is predominantly found in humans, it is also found in animals [32] and so this indicates the possibility of zoonotic transmission in the region.

The phylogeny also showed genetic variation within isolates of assemblage B, however, some isolates were genetically related despite their variation in the BLAST analysis. Seven isolates were grouped into the following sequences: BI (HQ397719.2), BIV (LO2116.1), BV (HQ666895.1), BVII (HQ666897.1), Hole H13 (KT948108.1) and HS98 (KC632554.1). Two isolates were grouped into sequence Swemon 200 (EU781015.1). The mixed assemblage sequences showed 96–99% identity with sequences registered in GenBank, which indicates that both assemblages in mixed infected isolates were apparently divergent.

The higher genetic heterogeneity observed within assemblage B than assemblage A isolates is supported by many previous studies [11, 16, 20, 30, 33]. Despite the various loci used among studies, most assemblage A isolates typed at TPI, GDH and βg belong to sub-assemblage AII [4, 9, 26], which was also observed in this study.

A study done in Sweden [34] indicated that sub-typing of assemblage A was easier than assemblage B. Similarly, in Rwanda [16], sub-typing of assemblage B at the TPI locus was unsuccessful. Most assemblage B sequences retrieved in this study showed frequent double peaks in chromatograms and determining the origin proved to be a challenging task. According to a previous study [35], the origin of double peaks was potentially linked to concurrent occurrence of different cysts in a sample or different allele in a cyst. The much higher allelic sequence divergence observed in the complete genome sequences of assemblage B [36] than in assemblage A [37], however, make allelic sequence divergence in a cyst more reasonable. The phylogeny also apparently placed all isolates in their respective clusters, reinforcing the absence of co-existed sub-assemblages. However, to ensure accuracy, multilocus analyses using both conserved and variable genes should be conducted, as assemblage B is genetically broad [38]. The present study showed the difficulty of assemblage B sub-typing at the TPI locus and stresses the need to develop new genotyping approaches.

### Risk factors associated with *G. duodenalis* infection

This study showed a higher rate of *G. duodenalis* infection in children and in municipal/tap water and open spring water consumers than in the other groups.

Studies conducted in Brazil [1], Malaysia [28], Uganda [29] and Mongolia [39] had findings consistent with this study and showed a higher rate of *G. duodenalis* infection in children than in adults. No differences in ages were, however, reported in Sao Tome and Principe [2] and Belgium [24].

Regarding sex, studies conducted in Brazil [1], Sao Tome and Principe [2], and England [9] had corresponding findings and showed no difference between *Giardia* infection and sex. In addition, in a rural area of Malaysia [28], Uganda [29], Yemen [31] and Portugal [40], similar findings were also reported.

The effect of individuals’ place of residence on *Giardia* infection was also consistent with study done in Yemen [31] and found no association between infection and residence, however, in Rwanda [16], it was reported that infection was associated with residence.

Even though a higher rate of infection was reported in municipal/tap and open spring water consumers than in river water consumers, this study did not conclude that the water sources in the region are intuitively contaminated. Various circumstances, such as limited follow-up, insufficient water treatment facilities and household level contamination could be attributable factors. The municipal/tap water supply was mostly sporadic and forced householders to store water for a long time, and this might have predisposed the water to contamination in the home environment. In addition, chlorination was the major, but sporadically used, water treatment method. Thereby, the high rate of infection could also be associated with this, as chlorination has a low effect on the disinfection of cysts. A study conducted in Spain [15] offers credence to this finding and showed that rapid filtration and chlorination treatment of water were insufficient to eliminate *Giardia* cysts. Open spring water sources were often unprotected or poorly protected, and located mostly in the rural areas of the region. The sources were mainly used for drinking, washing, watering domestic animals, and conducting different holiday celebrations and religious activities. These might predispose sources to contaminate. There was also no contamination prevention, water quality assurance follow-up and water treatment facilities applied to these sources.

In this region, some of the rivers were located near towns and were visibly contaminated, however, the rivers that were used for drinking were located far from where frequent anthropogenic activities were conducted and where domestic animals had contact. Supporting this finding, a study in Brazil demonstrated the absence of *Giardia* cysts in samples from 28 different rivers [27], however, a study done in northern Spain [15] found the highest number of *Giardia* cysts from river water. Remarkably, studies done in Sao Tome and Principe [2] and in Uganda [29] showed the absence of an association between *G. duodenalis* infection and drinking water sources. In general, the differences observed among studies could lie in the type of water treatment facilities used, seasons of sample collection, location of water sources and in the different activities carried out around the sources. Ethiopia is a low-income tropical country where different risk factors exacerbate the transmission of parasites. Thus, poor environmental sanitation [2], zoonotic transmission [14, 23], anthroponotic transmission [31], lack of education [40] and other unidentified factors could play important roles in the prevalence of *G. duodenalis*.

In this study, the genotypes of *G. duodenalis* were determined using a single TPI locus from one-time taken stool samples. This might have underestimated the true prevalence of *G. duodenalis* infection in the region. In addition, only some risk factors were investigated. Multilocus genotyping analysis would give a better genetic resolution. Investigating other risk factors may also help to better elucidate the epidemiological setting of *G. duodenalis* infection.

## Conclusions

Genotyping of *G. duodenalis* is a useful approach for understanding the diversity, transmission dynamics and infection risk factors of the parasite.

Infection with *G. duodenalis* was found to be considerably high in asymptomatic individuals in Southern Ethiopia. The assemblages that caused infection were similar to other areas, but their diversity showed some variations. Although TPI is a discriminatory genetic marker for sub-typing assemblage A, it is less valuable for assemblage B. In addition, determining the origin of mixed assemblage infections and double peaks observed in most isolates of assemblage B was challenging.

This study highlights the need for further multilocus genotyping studies and the development of new molecular approaches. The asymptomatic individuals in this region were unknown reservoirs of the parasite and a region-level screening programme would help to minimise transmission. The study also recommends health education of residents and water treatment service improvements in the region.

## Additional file

Additional file 1: Multiligual abstract in the six official working languages of the United Nations. (PDF 740 kb)

## Abbreviations

BLAST: Basic local alignment search tool
bp: Base pair
PCR: Polymerase chain reaction
TPI: Triosephosphate isomerase
