# Supplementary material for: Molecular epidemiology of Giardia duodenalis infection in humans in Southern Ethiopia: a triosephosphate isomerase gene-targeted analysis
Source: Infect Dis Poverty. 2018 Mar 5;7:17. doi: 10.1186/s40249-018-0397-4 (PMC5836388; doi:10.1186/s40249-018-0397-4)
Supplement: Supplementary file 1 — Multiligual abstract in the six official working languages of the United Nations. (PDF 740 kb) [file 40249_2018_397_MOESM1_ESM.pdf]

Translation of the abstract into the six official working languages of the United Nations

دراسة وبائية جزيئية لعدوى الجياردية المعوية في البشر في جنوب إثيوبيا: تحليل إيزوميراز ثلاثي الفوسفات مستهدف للجينات

منجيستو داميتي، زيليك ميكونن، تاديسي جيتاهون، دانتي سانتياجو، *لوك ليتز*

الملخص

الخلفية: *الجيارديا المعوية* هي طفيلي يتكون من تجمعات متميزة متعددة وراثيا. ويتسبب هذا النوع في أزمة صحية كبرى في الدول النامية. في حين ان التنوع الجزيئي، وديناميات الانتقال وعوامل الخطر لهذا النوع من الطفيليات لا يزال مجهولاً في هذه الدول. أجريت هذه الدراسة لتحديد الوبائيات الجزيئية لعدوى *الجياردية المعوية* في الأفراد الذين يعانون من أعراض في *جنوب إثيوبيا*.

منهجية البحث: بعوضة الملاريا الصينية من مارس إلى يونيو 2014، تم جمع عينات براز أخذت للتو من 590 فردا مختارين بشكل عشوائي. تم جمع البيانات الاجتماعية والديموجرافية باستخدام استبيان منظم تم اختباره مسبقا. تم إجراء التنميط الجيني باستخدام تفاعل سلسلة البلمرة وتسلسل الحمض النووي لإيزوميراز ثلاثي الفوسفات المبني على الجينات. تم تحديد هوية الوراثية والقرابة للعزلات باستخدام أداة تقصي التراتب الموضعي الأساسي وتحليل النشوء والتطور. كما تم تحليل عوامل الخطر المرتبطة بعدوى *الجياردية المعوية* باستخدام نماذج الانحدار اللوجستي ثنائي ومتعدد الحدود.

النتائج: أظهرت النتائج أن 18,1٪ (509/92) من الخاضعين للدراسة قد أصيبوا بالجياردية المعوية. من بين العزلات، تم تصنيف 35.9٪ (92/33) و 21.7٪ (92/20) في مجموعتين فرعيتين أ وب، على التوالي، في حين أظهر 42.4٪ (92/39) عدوى مختلطة من أ وب. معظم عزلات المجموعة أ (94٪، 33/31) كانت مطابقة بنسبة 100٪ للتسلسلات المسجلة في بنك الجينات، والتي كانت الأغلبية منها تابعة للمجموعة الفرعية أ II. ومع ذلك، فإن التباين الوراثي العالي وتواتر القمم المزدوجة جعل التنميط الجيني الفرعي للمجموعة ب أكثر إشكالية وكانت 20٪ فقط (20/4) من العزلات مطابقة 100٪ مع التسلسلات. وأظهرت عوامل الخطر فيما يتعلق بالعمر (*P*=0.032) ونوع مصدر مياه الشرب (*P*=0.003) على حد سواء ارتباطا كبيرا مع وقوع عدوى *الجياردية المعوية*.

الاستنتاجات: أوضحت هذه الدراسة توطن *الجياردية المعوية* في *جنوب إثيوبيا*. كانت العدوى بالمجموعة أ أكثر تواترا من المجموعة ب، وكان معدل الإصابة أعلى لدى الأطفال وبين مستهلكي المياه المنزلية/مياه الصنبور ومياه الينابيع المفتوحة عن المجموعات الأخرى. كانت عملية التصنيف الفرعي للمجموعة ب وتحديد أصل القمم المزدوجة صعبة. وتؤكد هذه الدراسة الحاجة إلى إجراء دراسات أكثر شمولية تركز على الأنواع الفرعية للمجموعة ب وأصل عدم التجانس.

Translated from English version into Arabic by Mahmoud Sami, proofread by DinaAbed, through

TRANSLATORS
WITHOUT BORDERS

埃塞俄比亚南部地区人群十二指肠贾第鞭毛虫（*Giardia duodenalis*）的分子流行病学研究：基于磷酸丙糖异构酶基因的分析

Mengistu Damitie, Zeleke Mekonnen, Tadesse Getahun, Dante Santiago, Luc Leyns

摘要

引言：十二指肠贾第鞭毛虫（*Giardia duodenalis*）是由多个基因型组成的复合体。该种贾第鞭毛虫给发展中国家带来了重大公共卫生危机。然而，在这些国家其分子多样性、传播动力学和风险因素均不确定。本研究旨在确定埃塞俄比亚南部无症状人群中十二指肠贾第鞭毛虫感染的分子流行病学特征。

方法：2014 年 3-6 月，收集随机抽取的 590 个无症状个体的新鲜粪便样本。采用预先测试过的结构化问卷收集社会人口学数据。采用基于磷酸丙糖异构酶基因的巢式 PCR 和 DNA 测序方法进行基因分型。应用基本的局部比对搜索工具和系统发育分析来明确遗传一致性和相关性。采用二元和多元 Logistic 回归模型分析十二指肠贾第鞭毛虫感染的危险因素。
结果：18.1%（92/509）的受试者感染了十二指肠贾第鞭毛虫，其中 A 型（assemblage A）和 B 型（assemblage B）的感染率分别为 35.9%（33/92）和 21.7%（20/92），42.4%(39/92)为 A 型和 B 型的混合感染。94%（31/33）的 A 型与 GenBank 登录的序列 100%相同，其中大部分属于亚基因 AⅡ型。然而，基因变异性和变异频率呈现出双高峰，使得 B 型的亚基因分型成为问题，仅有 20%（4/20）的分离株与 GenBank 序列的一致度达到 100%。年龄（*P*=0.032）和饮用水源类型（*P*=0.003）与十二指肠贾第鞭毛虫感染显著相关。
结论：本研究明确了埃塞俄比亚南部地区有十二指肠贾第鞭毛虫流行。A 型较 B 型的感染率更高，儿童、市政/自来水和开放泉水的消费者感染率高于其他组。B 型的亚基因分型以及确定双峰的来源具有一定挑战性。本研究表明，需要进一步开展更广泛的研究，需重点研究 B 型的亚基因分型和异质性的起源。

Translated from English version into Chinese by Translated by Xue-Jiao Teng, edited by Pin Yang

CDC CHINESE CENTER FOR DISEASE CONTROL AND PREVENTION
NATIONAL INSTITUTE OF PARASITIC DISEASES

Épidémiologie moléculaire des infestations à *Giardia duodenalis* dans les populations humaines du sud de l'Éthiopie: analyse ciblée du germe de la triose-phosphate isomérase

Mengistu Damitie, Zeleke Mekonnen, Tadesse Getahun, Dante Santiago, Luc Leyns

Résumé
Contexte: *Giardia duodenalis* est un complexe d'espèces composé de multiples assemblages génétiquement distincts, à l'origine d'une crise majeure de santé publique dans les pays en voie de développement. La diversité moléculaire, la dynamique de transmission et les facteurs de risque de ces espèces sont pourtant encore mal déterminés dans les pays en question. La présente étude avait pour but de déterminer l'épidémiologie moléculaire des infestations par *G. duodenalis* chez des sujets asymptomatiques dans le sud de l'Éthiopie.
Méthodes: Entre mars et juin 2014, nous avons recueilli extemporanément des échantillons de selles auprès de 590 sujets choisis au hasard, dont les données sociodémographiques ont été relevées à l'aide d'un questionnaire structuré testé au préalable. Le génotypage a été effectué par réaction en chaîne à la polymérase emboîtée visant le gène de la triose-phosphate isomérase et par séquençage de l'ADN. L'identité génétique et les liens d'apparentement des isolats ont été déterminés à l'aide de l'outil de recherche d'alignement local basique et de l'analyse phylogénétique. Les facteurs de risque d'infestation par *G. duodenalis* ont été analysés au moyen de modèles de régression logistique binaire et multinomiale.
Résultats: Les résultats montrent que 18,1 % des sujets de l'étude (92 sur 59) étaient infestés par *G. duodenalis*. Parmi les isolats, 35,9 % (33 sur 92) appartenaient à l'assemblage A et 21,7 % (20 sur 92) à l'assemblage B, tandis que 42,4 % (39 sur 92) présentaient une infestation mixte par les sous-

types A et B. La plupart des isolats de l’assemblage A (94 %, 31 sur 33) étaient identiques à 100 % aux séquences stockées dans GenBank, dont la majorité appartenaient au sous-assemblage AII. En revanche, la grande variabilité génétique et la fréquence des doubles pics ont posé des problèmes pour le sous-typage de l’assemblage B, et seuls 20 % des isolats (4 sur 20) concordaient à 100 % avec les séquences. Les facteurs de risque de l’âge (*P* = 0,032) et de la provenance de l’eau de boisson (*P* = 0,003) étaient associés de façon significative à la présence d’une infestation par *G. duodenalis*.

Conclusions: Cette étude a confirmé la présence endémique de *G. duodenalis* dans le sud de l’Éthiopie. L’assemblage A était plus fréquemment présent que l’assemblage B et le taux d’infestation était plus élevé chez les enfants et les consommateurs d’eau du robinet (municipale) et d’eau de sources à l’air libre que dans les autres groupes. Nous avons rencontré des difficultés dans le sous-typage de l’assemblage B et la détermination de l’origine des doubles pics. La présente étude confirme la nécessité d’autres études inclusives, axées sur les sous-types de l’assemblage B et sur l’origine de l’hétérogénéité.

Translated from English version into French by Suzanne Assenat, proofread by Nadine Verdier, through

TRANSLATORS
WITHOUT BORDERS

Молекулярная эпидемиология инфекции *Giardia duodenalis* у людей в Южной Эфиопии: анализ, нацеленный на определение генов триозофосфатизомеразы

Менгитсу Дамити (Mengistu Damitie), Зелеке Мекконен (Zeleke Mekonnen), Тадессе Гетахун (Tadesse Getahun), Данте Сантьяго (Dante Santiago), Люк Лейнс (Luc Leyns)

Реферат

Основная информация. Инфекция *Giardia duodenalis* представляет собой комплекс видов, состоящих из множества генетически различных составных частей. Ее виды являются причиной значительных кризисных ситуаций в государственном здравоохранении развивающихся стран. Однако молекулярное разнообразие, пути передачи и факторы риска для видов *Giardia duodenalis* не определены в этих странах. Это исследование проведено с целью определить в Южной Эфиопии молекулярную эпидемиологию *G. duodenalis* у лиц, не имеющих симптомов инфекции.

Методы. С марта по июнь 2014 г. собирали свежие образцы кала у 590 людей, отобранных случайным образом. Социальные и демографические данные собирали при помощи предварительно тестированного структурированного опросника. Проведено генотипирование методом гнездовой полимеразной цепной реакции для выделения генов триозофосфатизомеразы и секвенирования ДНК. Генетическая идентичность и родство изолятов определяли при помощи средства поиска основного локального выравнивания и филогенетического анализа. Анализ

связанных с инфекцией *G. duodenalis* факторов риска проведен с применением моделей бинарной и мультиноминальной логистической регрессии.

Результаты. Полученные результаты показали, что 18,1% (92/509) субъектов исследования были инфицированы *G. duodenalis*. Полученные от них изоляты 35,9% (33/92) и 21,7% (20/92) были распределены на подтипы с комплектами А и В соответственно, и у 42,4% (39/92) обнаружена смешанная инфекция, имеющая подтипы А и В. Большинство изолятов с комплектами А (94%,31/33) были на 100% идентичны последовательностям, зарегистрированным в GenBank; среди этого количества преобладали подтипы комплекта AII. Однако значительная генетическая вариабельность и частота двойных пиков привели к трудностям при генотипировании подтипов с комплектами В, и только 20% (4/20) изолятов на 100% совпадали с последовательностями. Оба фактора риска: возраст (*P*=0,032) и вид источника воды для питья (*P*=0,003) имеют статистически значимую связь с числом случаев инфекции *G. duodenalis*.

Выводы. Это исследование установило эндемичность инфицирования *G. duodenalis* в Южной Эфиопии. Инфекция, вызванная подтипом с комплектом А, встречается чаще, чем вызванная подтипом с комплектом В. Показатель инфицированности был выше у детей и лиц, пользующихся муниципальными/общественными и открытыми родниковыми источниками питьевой воды, по сравнению с другими группами. Изучение подтипов с комплектом В и определение происхождения двойных пиков было проблематичным. Настоящее исследование подтверждает необходимость дальнейших инклюзивных исследований, при их проведении следует обратить внимание на подтипы с комплексом В и происхождение гетерогенности.

Translated from English version into Russian by Ann Nosova, proofread by Natalia Potashnik, through

La epidemiología molecular de la infección por *Giardia lamblia* en humanos en el Sur de Etiopía: Un análisis apuntado a un gen de la triosa fosfato isomerasa

Mengistu Damitie, Zeleke Mekonnen, Tadesse Getahun, Dante Santiago, Luc Leyns

Resumen

Antecedentes: El *Giardia lamblia* es un complejo de especies que consta de múltiples ensamblajes de distinto tipo genético. La especie supone una grave crisis en la salud pública de los países en desarrollo. No obstante, no se ha determinado la diversidad molecular, la dinámica de transmisión ni los factores de riesgo de la especie en estos países. Este estudio se ha realizado para determinar la epidemiología molecular de la infección por *Giardia lamblia* en individuos asintomáticos en el Sur de Etiopía.

Metodología: Desde marzo a junio de 2014, se recogieron muestras de heces frescas de 590 individuos seleccionados al azar. Se reunió información sociodemográfica a través de un cuestionario estructurado probado previamente. La genotipificación se realizó usando la reacción en cadena de polimerasa

anidada basada en genes de la triosa fosfato isomerasa y la secuenciación de ADN. La identidad genética y la relación entre las cepas aisladas se determinaron mediante la herramienta de búsqueda de alineaciones locales básicas (Basic Local Alignment Search Tool) y el análisis filogenético. Los factores de riesgo asociados a la infección por *Giardia lamblia* se analizaron mediante modelos de regresión logística multinomiales y binarios.

Resultados: Los resultados demostraron que el 18,1% (92/509) de los pacientes se infectaron por *Giardia lamblia*. Entre las cepas aisladas, un 35,9% (33/92) y un 21,7% (20/92) se agruparon en los ensamblajes A y B, respectivamente, mientras que el 42,4% (39/92) presentó infecciones mixtas del tipo A y B. La mayoría de las cepas aisladas del A (94%, 31/33) fueron 100% idénticas a secuencias registradas en GenBank, donde la mayoría pertenecía al subensamblaje AII. Sin embargo, la alta variabilidad genética y la frecuencia de los picos dobles dificultó la subgenotipificación del ensamblaje B y solo el 20% (4/20) de las cepas aisladas coincidió un 100% con las secuencias. Los factores de riesgo de la edad (P=0,032) y la procedencia del agua para beber (P=0,003) dejaron en evidencia una significativa relación con la aparición de la infección por *Giardia lamblia* .

Conclusiones: Este estudio estableció la endemia de *Giardia lamblia* en el Sur de Etiopía. La infección del ensamblaje A fue más frecuente que el B y la tasa de infección fue más alta en niños y en consumidores de agua local/de la canilla y de manantial abierta que en los demás grupos. La subtipificación del ensamblaje B y la determinación del origen de los dobles picos fue compleja. Este estudio confirma la necesidad de realizar más estudios inclusivos enfocados en los subtipos del ensamblaje B y el origen de la heterogeneidad.

Translated from English version into Spanish by Constanza DN, proofread by soledadlescano, through

TRANSLATORS WITHOUT BORDERS
